# DEMENTIA IN INCARCERATED SETTINGS: MAKING A DIFFERENCE IN OHIO'S PRISON SYSTEM

**DOI:** 10.1093/geroni/igac059.922

**Published:** 2022-12-20

**Authors:** Margaret Sanders

## Abstract

The number of individuals living in incarcerated settings with dementia will double by 2030 and triple by 2050. Older adults are the fastest growing demographic in incarcerated settings and age is the number one risk factor for dementia. The HRSA-sponsored Geriatric Workforce Enhancement Program, Ohio Council for Cognitive Health, Benjamin Rose Institute on Aging and the Ohio Department of Rehabilitation and Correction (ODRC) worked together to develop Dementia Friends for Incarcerated Settings to impact the lives of those living with dementia within the correctional setting. This pilot furthered the development of this Dementia Friends programming and measured ODRC staff knowledge/attitudes about dementia, as well as uncovered the experiences and needs of staff within the ODRC. Online Dementia Friends sessions were conducted in all 27 Ohio prisons with voluntary, anonymous pre- and post-session surveys based on the Brief Tool for Dementia-Friendly Education and Training Sessions developed to measure the impact of dementia-friendly education efforts on knowledge, attitudes/perceptions, and interacting with people living with dementia and their caregivers. The results of this pilot underscored the need, usefulness and applicability of dementia education throughout the ODRC system, indicating teaching effective communication techniques within a correctional facility are helpful for those working within such settings. The results indicate benefits on both a personal and professional level. These sessions provide a starting point for potential future educational sessions to serve all members of the correctional sector. Future proposed projects will focus on people living in incarcerated settings and those transitioning back into the community.

